# Genetic Evaluation for Hereditary Cancer Syndromes Among African Americans: A Critical Review

**DOI:** 10.1093/oncolo/oyab082

**Published:** 2022-03-05

**Authors:** Ambreen Khan, Charles R Rogers, Carson D Kennedy, AnaMaria Lopez, Joanne Jeter

**Affiliations:** 1 Family Cancer Assessment Clinic, Huntsman Cancer Institute, Salt Lake City, UT, USA; 2 Department of Family and Preventive Medicine, University of Utah School of Medicine, USA; 3 Sidney Kimmel Cancer Center, Thomas Jefferson University, Philadelphia, PA, USA

**Keywords:** hereditary cancer syndromes, health disparities, African-American population, genetic testing, precision medicine

## Abstract

While hereditary cancer syndromes have been described and studied for centuries, the completion of the human genome project fueled accelerated progress in precision medicine due to the introduction of genetic testing in the 1990s, creating avenues for tailored treatments and medical management options. However, genetic testing has not benefited everyone equitably, with nearly all of the published work based on individuals of non-Hispanic White/European ancestry. There remains a gap in knowledge regarding the prevalence, penetrance, and manifestations of common hereditary cancer syndromes in the African-American population due to significant disparities in access and uptake of genetic testing. This review summarizes the available literature on genetic testing for breast, colon, and prostate cancers in the African-American population and explores the disparities in access to genetic testing between non-Hispanic White and African-American patients. This article also addresses the barriers to genetic testing and discrepancies in the uptake of recommendations for hereditary cancer syndromes in the African-American population when compared with non-Hispanic Whites. The review offers practice implications for many healthcare providers and demonstrates gaps in the existing knowledge to be addressed in future studies to help eliminate the persisting health disparities faced by the African-American population.

## Teaser

The two-fold purpose of this review is to summarize the literature on disparities in genetic evaluation for hereditary breast, colorectal, and prostate cancer syndromes in the African-American population in the United States and to describe areas of study and outreach in this medically underserved population. Barriers to genetic testing are addressed and interventions to improve the utilization of cancer genetics services are highlighted.

**Implications for Practice**

Genetics and genetic testing have been historically used against marginalized individuals in healthcare settings. Gaining trust from historically excluded populations such as African Americans is pertinent for clinicians/healthcare providers to achieve equitable health outcomes. This review summarizes the existing gaps in access to and uptake of genetic testing among African-American individuals, allowing healthcare providers to reflect on implicit and explicit biases that persist in the field of genetics. Since barriers to access exist at different entry points to care for African-American individuals, this article provides recommendations to address these barriers as well as outlines facilitators to increase access.

## Introduction

Hereditary cancer syndromes have been described since the 1800s, prompted in part by increased cancer prevalence observed in prominent families such as those of Napoleon and Madame Z.^1^ Genetic testing for many of these syndromes has been available since the 1990s. However, 30 years later, due to significant disparities in genetic testing access and uptake, relatively little is known about the unique prevalence, manifestations, and penetrance of these inherited syndromes in the non-Hispanic (NH) Black/African-American population (for the purpose of this article, we use African American as an inclusive term for individuals of African descent as well as individuals who identify as Black, with or without African lineage). Since the advent of genetic testing, facilitated by the completion of the Human Genome Project, most published data in this area have been based on populations of European ancestry.^2^ The 2-fold purpose of this review is to (1) summarize the available literature on disparities in genetic evaluation for hereditary breast, colorectal, and prostate cancer syndromes in the African-American population in the US and (2) describe further areas of study and outreach in this medically underserved population. We also address barriers to genetic testing and highlight interventions to improve the utilization of cancer genetics services in this population.

## Hereditary Breast Cancer

Breast cancer is the second most common cancer among African-American women; 1 in 9 African-American women, compared with 1 in 8 NH White (NHW) women, will be diagnosed with breast cancer.^3^ Incidence rates of breast cancer under the age of 45 years are 16% higher among African-American women than among NHW women (incidence rate ratio [IRR] = 1.16; 95% confidence interval [CI] = 1.10-1.23), and mortality from breast cancer is 42% higher in African-American patients than in NHW patients.^4^ The causes of these differences in outcomes are multifactorial and are partly attributable to a more advanced stage at diagnosis and unfavorable tumor characteristics such as triple-negative disease, high tumor grade, and inflammatory carcinoma.^3-7^

Genetic counseling and testing can identify hereditary cancer risks that have valuable implications for prevention and treatment. In the CARRIERS consortium, a group of over 28 000 participants, the prevalence rate for a pathogenic variant (PV) in one of the 12 breast cancer susceptibility genes was 5.65% for African-American women with breast cancer compared to 5.06% for NHW women with breast cancer (*P* = .12).^8^ Rates of genetic testing for individuals who meet guidelines for *BRCA1/2* testing remain sub-optimal across all populations despite access and insurance coverage.^9^ However, compared with other races and ethnicities, African-American patients with breast cancer have lower rates of referral for genetic evaluation.^10-15^ Additionally, African-American women with breast cancer are more likely than NHW women to have PVs in *BRCA2* (1.80% vs 1.24%; *P* = .005) and *PALB2* (1.01% vs 0.40%; *P* < .001).^8^ Ademuyiwa et al (2019)^11^ found that 41.2% of African-American women who were eligible for *BRCA1/2* mutation testing according to National Comprehensive Cancer Network (NCCN) guidelines did not receive it as part of their routine care. Healthcare providers were 16 times less likely to discuss genetic testing with African Americans than with their NHW counterparts.^16^ Similarly, Armstrong et al (2005)^10^ found that African Americans were significantly less likely than NH Whites with comparable cancer family histories to be referred for genetic counseling (odds ratio [OR], 0.22). McCarthy et al (2016)^13^ reported that African-American women’s physicians were less likely to recommend *BRCA1/2* testing (OR, 0.38; 95% CI, 0.32-0.45; *P* < .001), a difference that persisted after adjustment for mutation risk, clinical factors, sociodemographic characteristics, and attitudes toward testing.

Despite the low referral rate, studies demonstrate that African-American patients with breast cancer are interested in genetic testing.^6,14,17^ Compared with NHW women, African-American women had more positive attitudes about the benefits of genetic testing but lower levels of knowledge about genetic testing for breast cancer risks.^6^ Peterson et al (2020)^14^ found that, although genetic counseling referral rates differed by race, uptake of genetic counseling services among African-American patients who were referred was not significantly different than uptake among NHW women. Therefore, low levels of genetic evaluation in the African-American population are less likely to be attributable to a lack of interest.

Another important metric is the uptake of genetic testing by African-American women with breast cancer when offered by genetic counselors. Peters et al^18^ found in 2004 that, after adjusting for awareness, African-American patients were less likely than NHW women to endorse the potential benefits of genetic testing due to concerns about the use of genetic tests for racial discrimination and the establishment of racial hierarchy (OR, 2.15, 95% CI, 0.15-4.03), which highlights the mistrust of the healthcare system. Even after minimizing test cost and other barriers to accessing genetic testing services among African-American women, Susswein et al (2008)^19^ found that African-American women were significantly less likely than their NHW counterparts to pursue *BRCA1/2* testing when it was offered (58% vs 71%; OR, 0.54, 95% CI, 0.34-0.85). Some of the lack of uptake may be due to a lack of health insurance coverage.^20^ However, in a subset of this study’s population, African-American women who were more recently diagnosed with breast cancer (<1 year) were more amenable to genetic testing than those with a more remote diagnosis (>1 year before a genetic evaluation) (71% vs 66%; OR, 1.58, 95% CI, 1.10-2.29). Because genetic testing can inform immediate medical management options for some patients, this difference in uptake between recently and remotely diagnosed groups highlights the importance of offering a timely genetic assessment.

When genetic testing is offered, the acceptance rate for such testing can influence medical management and cascade testing for family members. Halbert et al (2006)^21^ evaluated acceptance rates for *BRCA1/2* test results among African-American women while considering cultural factors such as communalism and spiritual beliefs in this population. Among women who were at an increased risk for carrying a *BRCA1/2* mutation, less than half completed pre-genetic testing education and counseling, and only about one-fifth of the overall sample received test results.^21^ African-American women who were less certain about their risk of developing breast cancer were 3 times more likely to receive *BRCA1/2* test results compared with women who were more certain about their risk.^21^ Such discrepancies in the receipt of results based on risk certainty may be improved by emphasizing the utility of testing for both the individual and her family members. Testing uptake may be increased by ensuring informed decision making, including a thorough understanding of relative risks, the testing process, and the advantages and disadvantages of testing.^17^ In particular, discussion of protections against genetic discrimination, such as the Genetic Information Nondiscrimination Act (GINA), should be emphasized.

Differences in the uptake of genetic testing and counseling by African-American women with breast cancer––with earlier studies showing underutilization^18,19,21^ and more recent data^14^ showing no significant differences compared with NHW women––may be explained by an increase in the availability and accessibility of such services as well as their decreasing cost. Equitable access to genetic services, including increased genetic education and awareness as well as appropriate referrals and insurance coverage, is key to the use of these services by African-American women.^8^

## Hereditary Colorectal Cancer

Although preventable and treatable with early detection screening, colorectal cancer (CRC) disproportionally affects the African-American community. CRC is the third most common cancer in African Americans and the third most common cause of cancer-related death in African-American men and women.^3^ Incidence rates of CRC are 19%-24% higher in African Americans than in NH Whites, with African-American men having disproportionately higher rates of CRC-related mortality than any other racial/ethnic group.^22^

CRC tends to present at earlier ages in African Americans than in NHWs, with African Americans being 4 years younger at presentation compared with NH Whites (*P* = .0012) and often presenting with a more advanced disease.^23,24^ Although early age of diagnosis can indicate a hereditary cause of cancer, reports are conflicting as to whether rates of hereditary CRC syndromes are higher in African Americans than in the NHW population. Hereditary non-polyposis colorectal cancer (HNPCC)—also known as Lynch syndrome—is the most common hereditary CRC syndrome among high-risk African Americans. Recent analysis of a large, nonselective, ancestrally diverse database of over 30 000 subjects has suggested that the prevalence of HNPCC is significantly higher in people of African ancestry (1 in 299) than in those of NHW (1 in 518) or Hispanic/Latino ancestry (1 in 634).^25^

Given the higher prevalence of HNPCC among African Americans, access to genetic counseling and genetic testing can significantly improve mortality and reduce late-stage diagnoses.^26,27^ In a study of patients referred to a high-risk CRC clinic, African Americans’ lack of knowledge of family cancer history was cited as a factor limiting these individuals’ access to genetic testing.^28^ Prior studies have reported that family-history gathering and test interpretation may also be more complicated in the African-American population. Kupfer et al (2006)^28^ found that among patients referred to their clinic, 18.9% of African Americans did not know their family history in their paternal lineage compared to 6.5% of NH Whites (*P* ≤ .05). However, a more recent study suggested that only about one-third (31.1%) of the general population in the US tends to have a thorough knowledge of their family history information.^29^

Abnormal immunohistochemistry (IHC) results on colon tumor testing are another pathway to referral for genetic testing. Studies show a similar rate of mismatch repair deficiency on IHC testing of colon tumors in African-American and NHW populations, and many cancer centers have introduced universal screening for HNPCC for all colon cancers.^30-32^ Muller et al (2018)^33^ found a lower rate of provider referral for genetic evaluation among African Americans compared with NH Whites, even for those with abnormal IHC results (17% vs 21% in NHW, *P* = .02). Although there was no between-group difference in attendance at genetics appointments, the uptake rate for genetic testing was lower among African Americans (6% vs 11% in NH Whites, *P* = <.01). This pattern of decreased rates of referral, similar rates of appointment uptake, and decreased rates of testing uptake for African Americans compared with NH Whites merits further study.

## Hereditary Prostate Cancer

Prostate cancer is the most common cancer diagnosis and the second most common cause of cancer-related death in African-American men. It occurs more frequently and at an earlier age in African Americans than in NH Whites. The prevalence of prostate cancer in African-American men is 1 in 7 and the average age at diagnosis is 63 years, compared with a prevalence of 1 in 9 and an average age at diagnosis of 66 years in NHW men.^3^ African-American men are also more likely to be diagnosed with aggressive disease (rate ratio 3.08-4.91).^34^ Although the mortality rate from prostate cancer has markedly decreased in recent years among both African Americans and NH Whites (and decreased more quickly for African Americans than for NH Whites between 2006 and 2015 [3]), 5-year mortality for African Americans remains 2.5-fold higher than for NHW men.^35^ The higher incidence and mortality for African-American men with prostate cancer compared with their NHW counterparts persists even after accounting for socioeconomic factors.^36,37^

Familial prostate cancer has been recognized for decades, but a lack of identified predisposition genes has historically limited the use of genetic testing for this condition in both African Americans and the general population. The 2017 Philadelphia Prostate Cancer Consensus Conference provided the impetus to expand germline testing in the US to all men with metastatic castrate-resistant prostate cancer.^38^ These guidelines recommended using the same criteria for genetic testing in African-American men as in other men until additional data on molecular differences in prostate cancer in African-American men are available to guide more-tailored medical management. Due to a continuing absence of data, this recommendation did not change in a 2019 guideline update.^39^

Although current reports on the uptake and practices of genetic testing specific to the African American prostate cancer population are lacking, earlier studies have attempted to address this question. In a 2002 study of interest in genetic testing for prostate cancer predisposition among 320 African-American men,^40^ an overwhelming majority of subjects (87%) responded that they would like to have a (then theoretical) genetic test for hereditary prostate cancer. This response did not vary by age, education, or family history. Additional reports from the African-American Hereditary Prostate Cancer study (also conducted before the expansion of the guidelines on germline testing for prostate cancer susceptibility) demonstrated low levels of prostate cancer-specific knowledge and low levels of prostate cancer screening in this high-risk cohort. In this group of African-American men who had at least 4 family members with prostate cancer, rates of digital rectal examination (DRE) and prostate-specific antigen (PSA) screening were lower than those in the general African-American population (DRE 35% vs 45%; PSA 45% vs 65%).^41,42^

Although the literature on the population-specific incidence rate of the *BRCA1/2* mutation is scarce, a preliminary study by Petrovics et al (2016)^43^ identified pathogenic mutations and variants of uncertain significance (VUSs) in 7.3% of African-American patients with prostate cancer versus 2.2% of NHW patients. It is known that, compared with non-carriers, carriers of germline *BRCA1/2* mutations can present with more aggressive disease and have a higher risk of recurrence and prostate cancer-specific mortality. Moreover, therapeutic clinical trials are increasingly using germline *BRCA1/2* mutation carrier status to determine participant eligibility. Further research is therefore needed on the incidence and prevalence of inherited prostate cancer susceptibility in African-American men compared with NHW men. In addition to its implications for familial cancer risks among African-American families, this knowledge could be pertinent to reducing disparities in prostate cancer treatment, which could in turn lead to a reduction in prostate cancer mortality rates among African-American men.

## Barriers to Testing and Interventions

Access to genetic counseling and appropriate genetic testing can have far-reaching implications in improving health and reducing health-related disparities in medically underserved populations. Although testing for multi-gene hereditary cancers is becoming increasingly accessible, if access to appropriate pre- and post-test genetic counseling is limited, medically underserved patients may face barriers to the appropriate interpretation of results, potentially widening the gap in their treatment or medical management when compared with populations that are not underserved.

Ndugga-Kabuye et al (2019)^44^ found that African-American patients with cancer who were offered genetic testing for the *BRCA1/2* and HNPCC genes were more likely than their NHW counterparts to be identified as having a VUS (18.8% vs 6.1%; *P* < .001). This disparity persisted among African-American patients when compared to NHW patients without a personal history of cancer, who were offered genetic testing for the *BRCA1/2* and HNPCC genes based on their family history (12.3% vs 5.8%; *P* < .001).^44^ VUS results can be challenging both for patients and for healthcare providers who are not genetics specialists, as they can represent either a benign human variation or a change causing an increased risk for cancer. When mismanaged, VUS results can lead to potentially unnecessary and invasive surveillance, surgery, or misinformed family planning.

The largest population allele frequency database, the Genome Aggregation Database (gnomAD) contains 141,456 unrelated individuals from several large-scale sequencing projects and is routinely used to help with variant interpretation and classification. This database lacks ancestral diversity, with 58.2% of individuals represented being of European (Finnish; non-Finnish European; Ashkenazi Jewish) ancestry versus 8.8% African or African-American ancestry. Structural barriers may exist for equitable research participation leading to excessive homogeneity of source samples. This gap in knowledge creates barriers to variant interpretation for African-American patients, leaving their genetic test results more prone to mismanagement.

In addition to the lack of data on the incidence and prevalence of gene mutations in African-American patients, genetic referral rates for African-American patients lag behind those of NHW patients, despite studies showing that African-American cancer patients are interested in this information. The prevalence of systemic and implicit biases within healthcare organizations continues to cause healthcare providers to act as gatekeepers for genetic testing, resulting in lower referral rates for genetic services in African Americans compared with NH Whites.

One common misperception that may affect referral rates is that genetic evaluation is not affordable for patients who are uninsured or underinsured. African-American patients consistently have lower insurance benefits than NHW patients,^45^ and patients with Medicaid have been found to receive less genetic counseling than patients who are privately insured,^46^ despite the increasing accessibility of multi-gene panel testing, which can be performed for an out-of-pocket cost of about $150 to $250. In addition, the major companies offering genetic testing for hereditary cancer syndromes have patient assistance programs or payment plans to help reduce or eliminate the cost associated with genetic testing.

Although uptake of genetic counseling appointments does not appear to differ by race or ethnicity, the literature suggests that uptake of genetic testing does. This may be related to the cost of genetic testing, as mentioned above, or maybe affected by mistrust of the medical community in the African-American population. Individual-level inhibitors to the completion of genetic testing for hereditary cancer risk may also stem from African Americans’ knowledge, attitudes, beliefs, awareness, and perceptions.^47^

Rogers et al found that educational levels, unfavorable attitudes toward research, lack of healthcare access, and the legacy of medical mistrust stemming from the Tuskegee Syphilis Study and perpetuated by lived experience were key barriers to the uptake of prostate cancer genetic testing and research participation by African-American men.^48^ A report on medical mistrust as a barrier to the uptake of genetic services^47^ concluded that “…*African Americans were less likely to endorse health benefits of genetic testing and more likely to believe that the government would use test results to label groups as inferior*.”

These perceived barriers can potentially be eliminated through education–focused interventions that are also culture-specific.^42^ When aiming to reduce inequities in cancer outcomes and screening among underserved and socially vulnerable populations, culturally tailored health educational interventions are a promising approach for the genetic evaluation space. For instance, Pal and colleagues (2010)^49^ found that culturally target visual aids married to phone-based genetic counseling was improved inherited breast and ovarian cancer knowledge among young African-American women with invasive breast cancer. However, some interventions to improve patient education about genetic services have proven more effective than others. As noted above, Halbert et al (2006)^21^ found that culturally tailored versus standard genetic counseling resulted in no difference in the uptake of genetic testing by African-American patients with breast cancer. Psychoeducation intervention is an approach that has been investigated to improve uptake of genetic services in breast cancer survivors.^50,51^ To eradicate barriers to genetic services referral and uptake among African Americans, further development of targeted interventions to increase awareness and trust while reducing stigma is warranted.

Other interventions to facilitate genetic services uptake have been studied in African-American and other medically underserved populations. The Prostate REACH study was an intervention for 64 medically underserved men, a majority of whom (*n* = 37) were African American. Most participants valued the patient navigator services and assistance with insurance barriers that the study offered. Community partnerships and organizational relations were fundamental to the program’s success. However, despite using a broad range of community- and media-based recruitment methods, recruitment of underserved men was difficult for this project and others.^52,53^ Despite these challenges, partnership with community leaders and groups remains a critical component of programs to decrease health disparities.

Alternative education methods may be another effective intervention, particularly to address geographical disparities. Computer-assisted methods, video methods, and possibly group education may be approaches to increase genetic education and reduce out-of-pocket expenses.^54^ Even before the COVID-19 pandemic, genetic counseling via telemedicine was well established,^54^ including audio-only technology, and is not inferior to in-person counseling; thus, telemedicine can play a key role in reducing barriers to genetic counseling services.^55,56^ Major genetic testing companies have aided in the collection of DNA specimens for testing through in-home phlebotomy programs and postage-paid saliva kits. Although these interventions were not used specifically to facilitate uptake among African Americans, they could be constructed to target this medically underserved population.

## Recommendations and Conclusion

The following are key recommendations based on this review:

1. Although medical mistrust by the African-American community remains a serious problem, studies have shown that African-American patients are interested in genetic evaluation. Providers should seek ways to ensure referrals are placed for genetic evaluation for all appropriate patients, equitably and regardless of race. Low- and no-cost genetic testing is available in the US for lower-income individuals who meet testing criteria, and patients should be referred for testing despite potential concerns about cost. Genetic counselors can help facilitate this process.
2. Systemic biases may be present in existing guidelines, which were developed using data from predominantly NHW populations. Guidelines for testing and management of hereditary cancer conditions should undergo review for disparity issues and consider the inclusion of a health equity expert in panel membership. Uptake of genetic evaluation and testing appears to be best when offered shortly after a cancer diagnosis; however, providers and genetic counselors should be aware that uptake of genetic testing may be overwhelming for patients dealing with a new cancer diagnosis and consider providing medical guidance and psychosocial support to facilitate patients’ informed decisions about genetic testing.
3. Studies of the incidence and prevalence of cancer susceptibility genes as well the uptake of genetic testing and high-risk management strategies in the African-American population are lacking and further research is needed in this area. Strategies to enhance racial/ethnic diversity of research participation should be actively pursued as an expected standard.
4. Medical terminology such as the word *Lynch* in *Lynch syndrome* may invoke unnecessary trauma for African-American patients, who associate this word with the public acts of racial terrorism that resulted in more than 4000 African Americans being lynched in the US between 1877 and 1950. Although the impact of *Lynch syndrome* as a term has not previously been studied, the use of more culturally sensitive language, such as the term *HNPCC* instead of *Lynch syndrome*, may be an important step toward building trust and acknowledging harm.
5. Community partnerships with organizations serving African Americans should be explored as potential outreach opportunities to increase awareness of genetic evaluation.^44^
6. Educational outreach to medical professionals must address engaging diverse populations and tailoring genetic counseling paradigms to African-American populations and those in underserved areas, focusing on increasing awareness of and access to genetic evaluation through telephone and video visits.

As precision medicine is rapidly incorporated into every area of medicine, genetic testing will continue to have practice implications for both patients and healthcare providers. Identifying and addressing knowledge gaps in this area through culturally literate communication and education tactics can facilitate genetic testing uptake. These efforts will contribute to eliminating the persistent health disparities plaguing African-American communities who continue to suffer disproportionately from breast, colorectal, and prostate cancers.

## Data Availability

The data underlying this article will be shared on reasonable request to the corresponding author.
